# Managing mental illness in Ghana: the state of commonly prescribed psychotropic medicines

**DOI:** 10.1186/s13033-016-0061-y

**Published:** 2016-04-05

**Authors:** Samuel Oppong, Irene A. Kretchy, Emelia P. Imbeah, Barima A. Afrane

**Affiliations:** Department of Pharmacy Practice and Clinical Pharmacy, School of Pharmacy, University of Ghana, Legon, Ghana

**Keywords:** Psychotropic medications, Antipsychotics, Antidepressant, Anticonvulsants, Anxiolytics, Hypnosedatives, Mental illness, Ghana

## Abstract

**Background:**

In Ghana, about 13 % of the adult population is estimated to be affected by mental health disorders of varying forms. In managing these patients, psychotropic medications are mostly employed. Since most of these conditions are chronic cases, the medications are consumed for prolonged periods of time. However, there exists an absence of information on efficacy, side effects, accessibility and prescription practices of psychotropic medication utilization from the viewpoint of the practitioners who are primarily involved in prescribing, dispensing and administering these medications.

**Method:**

Qualitative study composed of semi-structured interviews were conducted with twenty three (23) participants from Accra psychiatry, Pantang and Ankaful hospitals. These were fifteen (15) nurses, six (6) clinicians and two (2) pharmacists. All interviews were recorded digitally and analyzed thematically.

**Results:**

The commonly prescribed psychotropic medications were grouped into four classes. These were antipsychotics, antidepressants, anticonvulsants and hypnosedatives. Although each facility had at least one drug belonging to each class, there were frequent shortages recorded across the board. Also, drugs were free when supplied by government, and expensive when obtained from outside. When subsidized, the average cost of a day’s supply of the most common antipsychotic was 4 % of the daily minimum wage. The procurement system for the medications was fraught with challenges such as inadequate financing, poor procurement practices and bureaucracies with the process which affected the availability and quality of medications.

**Conclusion:**

The commonly prescribed psychotropic medications are in conformity with the recommendations of the WHO guidelines and the standard treatment guidelines of Ghana. However, the accessibility and quality of medications in the sector are inadequate. To improve mental health services in the country, it is important to ensure the adequate and regular provision of quality medicines in the mental health sector.

## Background

Worldwide, mental health disorders account for 14 % of the burden of diseases [1]. Similarly, the prevalence of mental health disorders in Ghana is 13 % of the adult population [2]. These disorders are of varying forms and may require numerous forms of care which could be either pharmacological or non-pharmacological [3, 4].

The mental health sector in Ghana hugely adopts the pharmacological method of managing mental illness due to the lack of personnel and resources for psychosocial therapies and rehabilitation [2, 5, 6]. Primarily, this involves the use of medicines, suitably referred to as psychotropic medications in sustaining patients. In Ghana, psychotropic medicines have become the mainstay of therapy.

Like other medications, psychotropic medications have undergone numerous phases of development which generally seek to improve upon the existing ones with regard to issues of efficacy, stability, side effects and specificity of actions. This has therefore accounted for the various generations of medications notably, the newer and older generations [7]. Ghana practices a hybrid system of combining both generations, in an imbalanced fashion, favoring the older generations because they are less expensive [8, 9]. These are the medications that have been implicated in much notoriety and their usage expected to be on the decline [10].

Despite the widespread use of the older generation and gradual infiltration of the newer generation into the system, less is known about the composite state of the medications especially from the viewpoint of the practitioners who are primarily involved in prescribing, dispensing and administering these medications. Issues regarding medications are often mentioned in studies that seek to examine other issues in the mental health sector bordering on policies, legislation and social framework of the sector [2, 5, 6, 8]. This current study therefore explored the psychotropic medications used in Ghana from the perspective of the practitioners along the areas of efficacy, side effects, accessibility and prescription practices.

## Methods

### Study site

The study was conducted in the three public psychiatric hospitals in the country; Ankaful Hospital (AH), Pantang Hospital (PH) and the Accra Psychiatric Hospitals (APH). The AH was built in June 1965 and currently has a bed capacity of 500 to serve the inhabitants of the Central, Western and Ashanti Regions of Ghana. The AH serves about 7788 out-patients annually. It has 323 staff members comprising 2 psychiatrists, 190 nurses, 1 occupational therapist, 1 psychologist and 129 other auxiliary staff. The PH manages patients with both physical and mental health conditions. Initially built with a 500 bed capacity, the hospital currently has 9 wards housing an average of 50 beds each. There is an average of 23,331 annual out-patient psychiatry cases and a total of 1337 in-patient cases. The hospital has 464 staff comprising nurses (316), pharmacists (1), psychiatrists (2), medical officers (1), psychologists (1), social workers (2), occupational therapist (1) and other auxiliary staff (140). The APH is situated in Adabakra, in the Greater Accra Region. It comprises 21 wards housing 478 in-patients and records an average of 120 out-patient attendances. It has 451 staff members which comprises nurses (343), pharmacists (2), psychiatrists (4), medical officers (4), psychologist (1), social workers (4) and 108 other staff (http://www.thekintampoproject.org).

### Participants

Participants for the study were selected based on convenience sampling. Specifically, participants engaged were nurses, clinicians and pharmacists. Clinicians herein mentioned referred to psychiatrists, physicians and medical assistants. A total of 23 participants were sampled for the study. This comprised fifteen nurses (5 from each hospital), six clinicians (2 from each hospital) and two pharmacists, one each from the Accra Psychiatric Hospital and Pantang Hospital. Prior to the commencement of the main study, a pilot study was conducted. This was undertaken involving three nurses and two clinicians from the Accra Psychiatric Hospital. The pilot study was to aid in identifying the possible constraints of the study procedure as well as in developing appropriate questionnaires for the study.

### Study tools

Based on available literature in the field and the aims of the study, an interview guide was developed. The interviews were semi-structured and each interview lasted an average of 30 min. Open ended questions were used to generate responses from the practitioners. Further probes and questions were used to illicit lucid responses and establish clarity.

### Data management

All interviews were recorded using a digital audio recorder. The recordings were then transcribed verbatim and analysed with the aid of thematic analysis. The transcripts were carefully perused and manually coded to generate a coding frame. The codes were grouped into basic themes. The basic themes thus developed were merged into organizational themes or subthemes. A group of the subthemes were arched into global themes. These three themes were used in generating a thematic network which aided in the analysis [11]. Observations and other non-verbal discourses were recorded as memos in a notepad.

### Ethics

Permission was obtained from the hospital authorities from the various institutions before the commencement of the study. All the participants gave informed written consent to participate in the study and to have the interviews recorded. Participants were assured of confidentiality in the analysis, processing, and presentation of results of the study. To ensure the information were anonymous and confidential, each participant was assigned an identification code which has been used in the presentation of the results. The participants are identified with their narratives using C, N, and P for clinicians, nurses and pharmacists respectively.

## Results

### Characteristics

The participants comprised clinicians (6), nurses (15) and pharmacists (2). Eleven out of the 23 participants interviewed were females with the majority (10) being nurses and one Clinician. Participants interviewed cut across the ranks of the various professions. The nurses included staff nurses (9), senior staff nurses (4) and nursing officers (1). There were 3 medical assistants, 2 physicians and a psychiatrist. One of the pharmacists was of senior pharmacist rank and the other, a deputy director.

### Themes

**Fig. 1:**
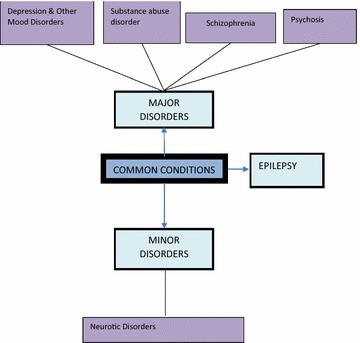
Common conditions *N* nurse, *C* clinician, *P* pharmacist.  Global theme,  Organizational theme,  Basic theme

**Fig. 2:**
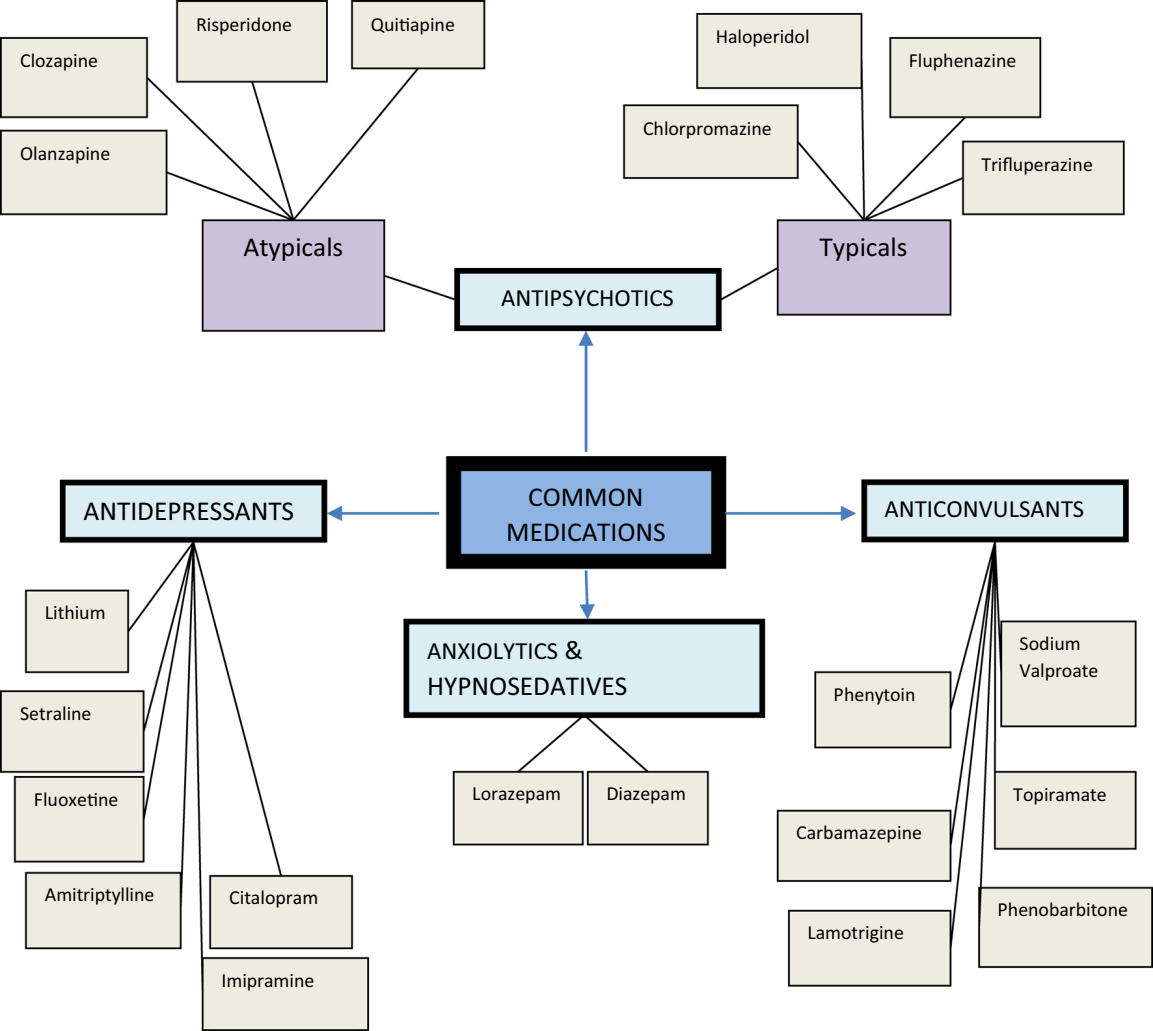
Common medications *N* nurse, *C* clinician, *P* pharmacist  Global theme,  Organizational theme,  Basic theme,  Codes

**Fig. 3:**
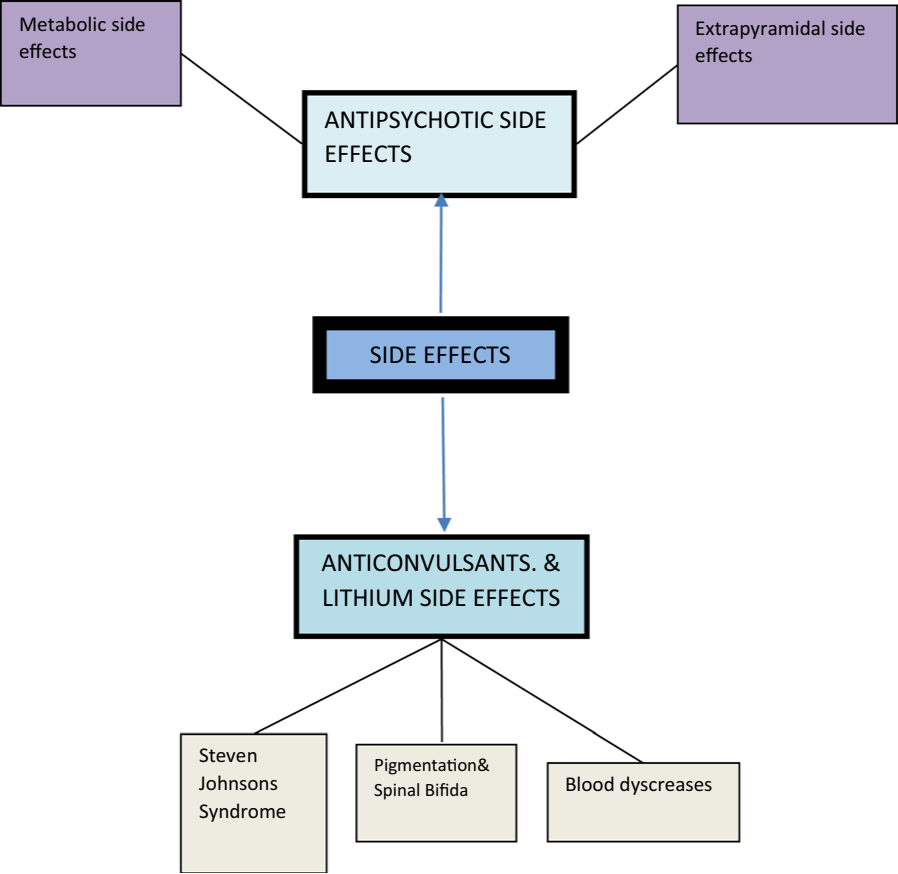
Side effects *N* nurse, *C* clinician, *P* pharmacist  Global theme,  Organizational theme,  Basic theme,  Codes

**Fig. 4:**
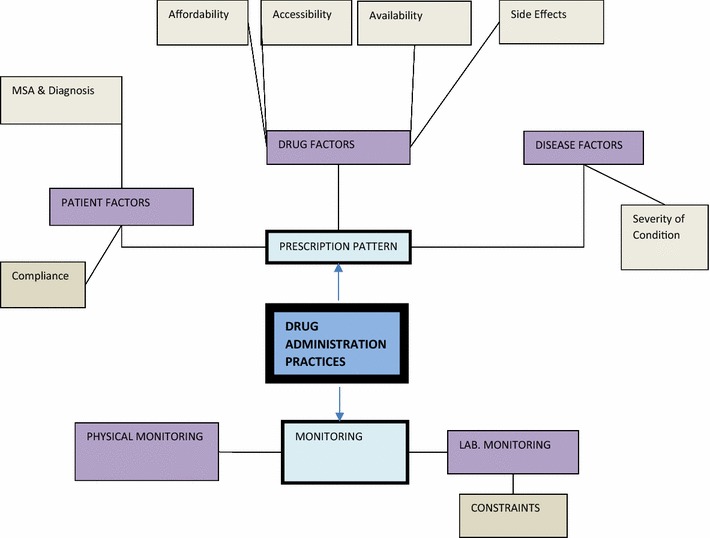
Drug administration practices *N* nurse, *C* clinician, *P* pharmacist  Global theme,  Organizational theme,  Basic theme,  Codes *MSA* mental state assessment

**Fig. 5:**
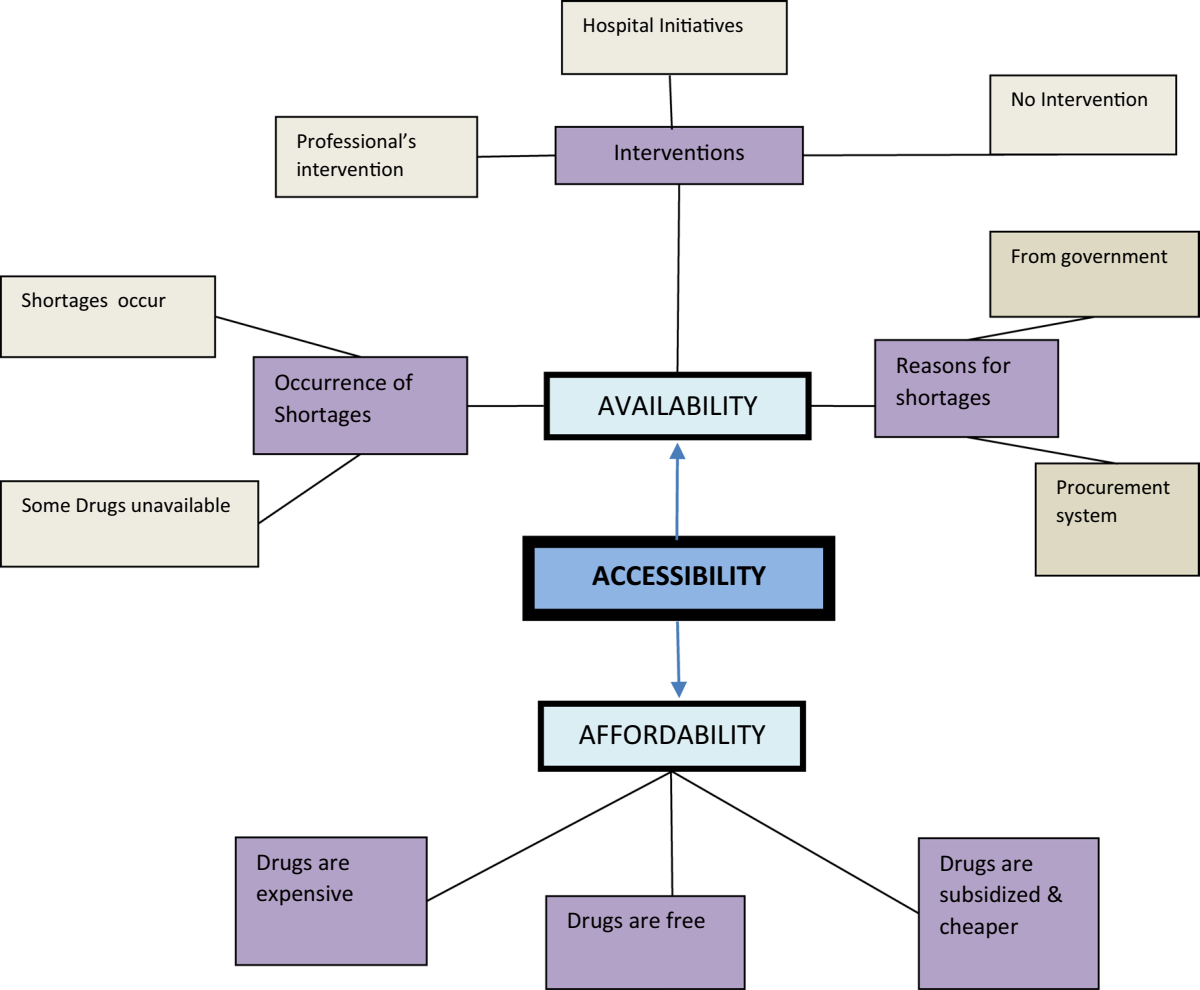
Accessibility *N* nurse, *C* clinician, *P* pharmacist  Global theme,  Organizational theme,  Basic theme,  Codes

**Fig. 6:**
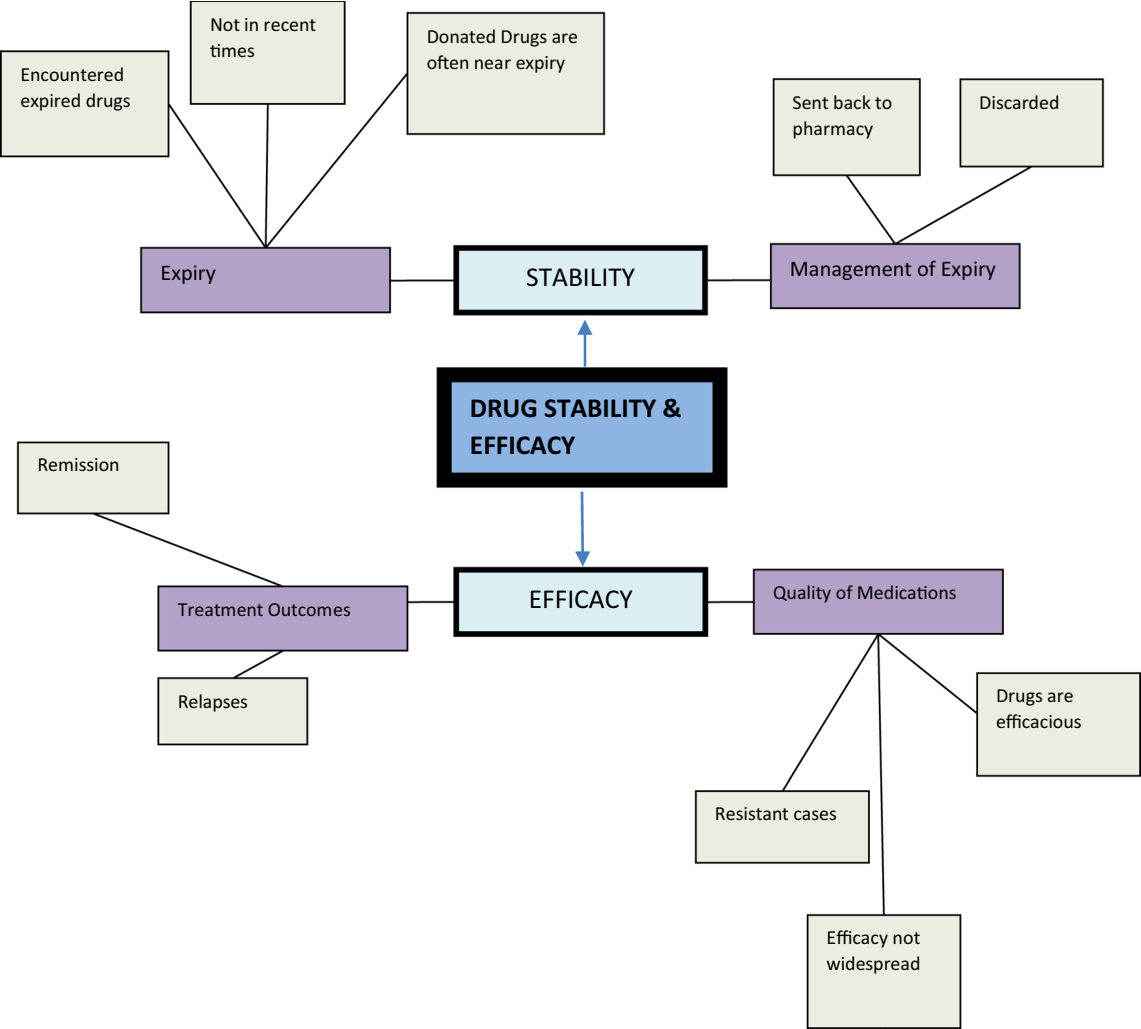
Drug stability and efficacy *N* nurse, *C* clinician, *P* pharmacist  Global theme,  Organizational theme,  Basic theme,  Codes

The analysis elicited the following themes: *Common conditions* (Fig. 1)*, Common medications* (Fig. 2)*, Side effects* (Fig. 3), *Drug administration practices* (Fig. 4)*, Accessibility* (Fig. 5) *and Drug stability and efficacy* (Fig. 6). The figures are diagrammatic representations of the main global, organizational basic themes. The basic themes are the textual concepts that were derived from the transcripts. They represented obvious thoughts, ideas or views that were expressed by the participants. The organizational themes arose from a cluster of basic themes and primarily sought to organize basic themes with similar concepts into a much more defined group. The global theme was the encapsulating theme, taking into account the organizational and basic themes which gave it a deeper meaning. Distinctions amongst these 3 were created using defined colour codes.

### Common conditions

The theme identified the various conditions presented at the psychiatric institutions. The common conditions presented varied across the wards since patients were admitted to specific wards based on presenting conditions. Two clinicians grouped psychiatric conditions into major and minor states. These classifications were based on the severity of presentation and the gravity of disturbance created within the individual and to the environment. Psychosis, depression and other affective disorders formed the major disorders while the minor ones were composed of generalized anxiety disorder, mild depression and obsessive compulsive disorders;

*We have the two major groups or types of mental illness which is the major mental illness, which is the psychosis and the minor one which is neurosis…….*(C1).

Besides these major groupings, there were a few other categorizations of the conditions; there were conditions referred to as the mood disorders which comprised hypomania, mania and bipolar disorders. Also, numerous forms of psychosis were presented; drug induced psychosis, neuropsychosis, undefined psychosis, post-partum psychosis and puerperal psychosis. Mental retardation or arrest of the mind was presented by two respondents as one of the conditions. Schizophrenia was the most mentioned condition. Conditions such as substance abuse, delirium, and dementia also came up in the responses. Although mentioned as a common clinical condition, epilepsy was seen by two respondents to be out of place in psychiatry;

*Normally it is said that epileptic cases should be seen by the general hospital but they have turned these patients to the mental hospital.* (C1).

### Common medications

Psychotropic medications were also known as psychoactive medications in the responses. The commonly prescribed medications were grouped into various categories often based on the conditions they were used to manage. Such groupings were common amongst the Clinicians and a few nurses while ten of the nurses gave a rough spread of medications when asked. The various groups of psychotropic medicines given were antipsychotics, antidepressant, anticonvulsants, anxiolytics and hypnosedatives.

Antipsychotics were said to be in two groups; the typical or first generation antipsychotics and then the atypical or second generation antipsychotics. Examples of the typical antipsychotics were given as chlorpromazine, haloperidol, trifluperazine and thioridazine. Olanzapine, risperidone, clozapine were examples of the atypical antipsychotics. Chlorpromazine (CPZ), marketed under the brand largactil was seen as the commonest of the typical antipsychotics and still used in current practice;

*Here we use olanzapine, haloperidol and risperidone ….but the commonly used ones on the ward are these 3 medications. And then sorry, the almighty CPZ*- *largactil.* (N8).

The atypical antipsychotics that emerged from the responses were olanzapine, clozapine, risperidone and quitiapine with olanzapine having the greatest frequency of use. Due to the minimal side effects of the atypical antipsychotics they served as better alternatives to the typical antipsychotics;

*Initially that is what they were using but it has more side effects than the new ones just introduced… like the olanzapines and the risperdones have mild side effects*. (N2).

Some respondents also referred to antidepressants as ‘psychic energizers’. They included amitryptilline, setraline, fluoxetine, lithium, imipramine and citalopram. While the term ‘antiepileptic’ was used interchangeably with ‘anticonvulsants’ in most responses, respondents identified carbamazepine, sodium valproate, phenobarbitone, phenytoin, topiramate and lamotrigine as the antiepileptic agents available.

Of these, carbamazepine (Tegretol^®^) and sodium valproate (Epilim^®^) had the highest frequencies. Besides being anticonvulsants they were noted as mood stabilizers by some of the respondents;

*……and we also have carbamazepine, it is a mood stabilizer and at the same time anticonvulsant.* (N12).

Diazepam and lorazepam were the commonly prescribed anxiolytics which was also known as the minor tranquilizers.

### Side effects

Respondents gave a myriad of side effects in relation to the consumption of the medications. Side effects to these medications were coded under antipsychotics, anticonvulsants and lithium. Antipsychotics were responsible for most of the side effects mentioned. Their side effects were grouped into extrapyramidal and metabolic side effects. The typical antipsychotics were noted for the extrapyramidal side effects while the atypicals were responsible for the metabolic side effects. The extrapyramidal side effects included dystonia dyskinesia, drooling, occulogyric crisis and twisting of neck while the metabolic disorders were said to include diabetes and weight gain. A clinician summed these in the switch from the older to the newer generation as;

*We were running away from extrapyramidal side effect but then we ended up with metabolic issues. They could have dyslipidemia, changes in their cholesterol, they could have diabetes because of problems with metabolism and sugars……..And then the weight gain is a problem…..They may have amenorrhoea or dysmenorrhoea which may affect fertility*. (C6).

Lithium carbonate was known to cause blood dyscrasia which was linked to its toxicity. Anticonvulsants were known to cause Steven Johnson’s syndrome and hypoencephalus or spinal bifida as recounted by two clinicians.

The principal management method for these side effects was the concurrent administration of counteractive drugs. These drugs were trihexyphenidyl (artane), benztropine and chlorpheniramine. Although rarely, a clinician reported switch of medications as a management method for the side effects;

*We take them off the medication; we stabilize them and move on to a safer drug*. (C5).

### Drug administration practices

This theme examined the various activities that are encompassed in drug administration. Emerging sub themes were the prescription pattern and drug monitoring.

The prescription pattern of the medications was ideally the call of clinicians. Nevertheless, most nurses provided information on prescription patterns while others referred it to the appropriate professionals. Generally, the prescription pattern was informed by Drug factors, patient factors and disease factors. Drug factors influencing the prescription pattern were primarily availability, affordability, and accessibility and drug side effects.

*Yea,..because anybody prescribing a drug, if you are a doctor and prescribing a drug you have to consider the availability of the drug, accessibility and its affordability. These three things should go into consideration….* (C1).

In Accra Psychiatric hospital, it was mentioned that daily bulletins of drugs in stock are issued from the pharmacy to all clinicians to aid in decision making.

The patient factors that affected prescription patterns were usually the diagnosis made through assessment of patients. Daily assessments collated through nurses’ reports were sent to the clinicians for review and necessary actions were then taken. Although the lack of availability of assessment tools for diagnosis was mentioned, the mental state assessment (MSA) was the tool often used to aid in diagnosis of some mental illnesses.

*Oh..clinical principles, you take a history, you examine, for us we do a mental state assessment and then based on those parameters you come by a diagnosis and then based on what the condition is you select a group of drugs or what the specific drug and appropriate for that condition.* (C5).

*In psychiatry we don’t have any test for people. It is just subjective. What the patient tells the doctor, the doctor just uses the DSM manual to try and categorize it. We don’t have any laboratory test to confirm, in this part of the world. But in advanced countries they are doing it.* (P2).

The majority of the respondents (21) admitted various forms of either physical monitoring or laboratory monitoring were undertaken. Physical monitoring included taking of vitals, the MSA and visual observations. These were regularly performed as reflected in the nurses’ responses.

Laboratory monitoring required the use of biochemical analysis (often blood) in measuring the levels and effects of some drugs in the body. It included the fasting blood sugar which was an indication for the diabetic effect of some drugs and the full blood count, which gave an indication of the blood cells depleting effects of some drugs.

*It is only clozapine that I understand it depletes the white blood cells so we do full blood count every 2* *weeks before we put any patient on it.* (C1).

Participants in all three institutions acknowledged the fact that these tests for monitoring were not routinely performed due to financial constraints and inadequate logistics.

*…….the truth of the matter is, ideally in giving these drugs, all of the drugs we have our baseline data; full blood count, Liver function test, blood urea nitrogen & creatinine clearance to monitor the renal function. These are basic but in practicality the issue is we don’t do it all. We don’t.* (C2).

### Accessibility

The accessibility of the psychotropic medications was discussed along two major facets; the availability and affordability of the medications. With regard to availability, twenty-two respondents admitted the existence of shortages. To some, shortages were seen to be rife all year round while others acknowledged shortages as a seasonal occurrence. These shortages were in relation to the absence or infrequent supply of some common medications. In such cases, patients were either asked to purchase medications themselves, were switched to alternative medications or were completely starved of medications until a supply arrived.

*Oh shortages! I will tell you it is a day to day battle. Today this is available tomorrow it is not. You cannot say for sure that. There is no guarantee that you write a medication today and tomorrow it is available.* (D5).

Reasons accounting for the shortages were directed at the office of the chief procurement officer of the psychotropic medications. Most respondents were ignorant of the factors responsible for the shortages but surmised poor funding from the government could be responsible for the shortages. The cumbersome nature of the procurement process for the drugs was also known to account for the shortages. The entire procurement process was said to be bureaucratic and that resulted in delays in drug delivery for as long as 2 years.

It was generally acknowledged that mental health services are free and as such patients do not pay for medications. In events of shortages, drugs became expensive when patients had to acquire from private pharmacies and relatively cheaper when bought from the pharmacies within the hospitals.

### Drug stability and efficacy

With regard to stability, the study primarily probed into issues of expiry and the management of such drugs when encountered. A few respondents admitted to have come across expired drugs in their facilities either in recent times, or in the past.

*We come across expired drugs…I called the attention of the in charge and he dealt with the expired drugs.* (N10).

Two major sources of expired medications were identified. First, failure of clinicians to prescribe a particular drug resulted in hoarding over a period of time beyond their shelf life. Secondly, some drug donations obtained from donor agencies were said to often be products that were near expiry and when not consumed within a period, eventually got expired. Such products were discarded.

In assessing efficacy, the treatment outcomes and quality of medications were looked at. Treatment outcome herein is defined as the response of the patients to therapy. Relapses were seen to be hugely common amongst patients. This response cut across institutions and professionals. The nature of the condition was partly responsible for relapse. Thus, relapses were inherent in the definition of mental disorders.

Also relapses were noted to be dominant amongst non-compliant patients who refused medications either as a result of lack of insight, side effects from medications or non-availability of medications;

*So most of them they relapse especially due to non*-*compliance of the drugs.* (C3).

The issue of unavailability accounted for relapses amongst patients on the wards besides other factors such as lack of family support when discharges were due, infrequent reviews by clinicians and the switch of medications.

*Some, as they are still on the ward they can relapse because they don’t have frequent reviews by their clinicians.* (N7).

## Discussion

Psychosis and schizophrenia were the common conditions existing in all three institutions. Due to the similarity in the clinical presentations of either of these conditions, respondents were likely to interchange the use of the words except in few cases of informed Clinicians who sought to differentiate between the two. The frequency of the conditions presented differed slightly from official records [2]. This could have been partly due to the captioning of most conditions under one major category of disease other than the fragmentation observed in official records. The term ‘mood disorders’ was generally used to encapsulate the terms bipolar, manic or hypomania.

The common medications prescribed clearly gave an indication of the prevalent conditions in the system. Although not the most ideal, there still is a pronounced prescription of the typical antipsychotics. Such has been the situation for years now, giving reasons of cost and availability. They were relatively easily available and cheaper to obtain. These factors to an extent undermined issues of side effects produced by these medications when prescribing. Gradually, there is an infiltration of the newer generation antipsychotics which have much patient tolerability. It is likely that as patient preference for the atypical medications increases, there will be a corresponding shift in the prescription pattern.

Amitriptylline, sertraline and fluoxetine were the antidepressants mentioned. Accordingly, these are the only preparations indicated for the management of depression in Ghana [12].

Most of the side effects mentioned such as dystonia, rolling of eyes and drooling of tongue are attributable to the typical antipsychotics. This called for regular co- prescribing of trihexyphenidyl to manage the side effects produced. It is necessary the long term economic and health viability of such practices be examined adequately to help inform stakeholders on the procurement of antipsychotics. The metabolic disorders produced by the newer generation medications were not hugely pronounced, possibly due to the low frequency in prescribing and better patient tolerability.

Although at least one medication belonging to each therapeutic class of medication was available, shortages were rampant. Shortages could therefore be seen as an inadequate supply of the medications to patients, or the restriction of the available medications to a minority that could either afford or access through other privileges. Besides the inadequate funding from government (which solely funds the mental health sector), hitches in the procurement system could have accounted for the shortages [13]. As such, the often available medications were the ones which were locally manufactured and less costly. By extension, local production of some of the medications is expedient in handling issues of shortages.

Prescribing of psychotropic medications bordered on professional competence. This was reflected in the varying depth of insights expressed by the various clinicians with regard to the conditions and the medications. The use of the mental status assessment (MSA) is a recognized tool which aids in diagnosing mental illness. The MSA is a structured, in depth guideline in assessing the mental state of an individual prior to diagnosis [14]. It focuses on observations made by Clinicians and responses inquired from individuals along certain core areas including appearance, speech, mood, thought, insight and attitude. This was not a much mentioned influence in prescribing possibly because irrespective of the diagnosis made the other factors had much influence on the prescription pattern. The availability and cost of the medications tied the Clinicians to very limited options. This has been responsible for a dominance of older generation drugs such as chlorpromazine and haloperidol in current practice. These are drugs which have pronounced sedative effects [15] and perhaps these sedative effects were harnessed to calm patients.

The study acknowledges the following limitations. First, the sample contained only two pharmacists which is a reflection of the huge deficit of pharmacists working in the public psychiatric hospitals in Ghana. Given the important role of the pharmacist in providing information on the actual dispensing of psychotropic medications in the psychiatric hospital, a larger number of pharmacists would have been appropriate. Second, although the study obtained information on the categories of prescribed psychotropic medications, triangulation of facts on the quantities of medicines utilized could potentially enhance the data. The information could be obtained through the inclusion of less subjective sources such as a review of prescription records from patient charts, and pharmacy inventory reports over a period of time.

## Conclusions

The study assessed the commonly prescribed psychotropic medications in Ghana from the perspective of the practitioners. The commonly prescribed psychotropic medications are in conformity with the recommendations of the WHO guidelines and the standard treatment guidelines of the country. However, the accessibility and quality of medications are inadequate. This is partly attributable to insufficient funds to support the sector and technical challenges by stakeholders in assessing the medications. The availability of medications in the sector is also stifled by the procurement system for the drugs. To improve mental health services in the country, it is important to ensure the adequate and regular provision of quality medicines in the mental health sector.
